# The Leukotriene Receptor Antagonist Montelukast Reduces Alpha-Synuclein Load and Restores Memory in an Animal Model of Dementia with Lewy Bodies

**DOI:** 10.1007/s13311-020-00836-3

**Published:** 2020-02-18

**Authors:** Julia Marschallinger, Barbara Altendorfer, Edward Rockenstein, Miriam Holztrattner, Julia Garnweidner-Raith, Nadine Pillichshammer, Iris Leister, Birgit Hutter-Paier, Katharina Strempfl, Michael S. Unger, Mansoor Chishty, Thomas Felder, Mary Johnson, Johannes Attems, Eliezer Masliah, Ludwig Aigner

**Affiliations:** 1 Institute of Molecular Regenerative Medicine, Paracelsus Medical University, Salzburg, Austria; 2 Spinal Cord Injury and Tissue Regeneration Center Salzburg (SCI-TReCS), Paracelsus Medical University, Salzburg, Austria; 3 Department of Neuroscience, School of Medicine, University of California San Diego, San Diego, USA; 4 QPS Austria GmbH, Neuropharmacology, Grambach, Austria; 5 Pharmidex, London, W1S 1RR UK; 6 Department of Laboratory Medicine, Paracelsus Medical University, Salzburg, Austria; 7 Institute of Neuroscience, Newcastle University, Newcastle upon Tyne, UK; 8 Austrian Cluster for Tissue Regeneration, Vienna, Austria

**Keywords:** Leukotrienes, autophagy, alpha-synulcein, Montelukast, neuroinflammation, dementia, cognition

## Abstract

Dementia with Lewy bodies (DLB) represents a huge medical need as it accounts for up to 30% of all dementia cases, and there is no cure available. The underyling spectrum of pathology is complex and creates a challenge for targeted molecular therapies. We here tested the hypothesis that leukotrienes are involved in the pathology of DLB and that blocking leukotrienes through Montelukast, a leukotriene receptor antagonist and approved anti-asthmatic drug, might alleviate pathology and restore cognitive functions. Expression of 5-lipoxygenase, the rate-limiting enzyme for leukotriene production, was indeed elevated in brains with DLB. Treatment of cognitively deficient human alpha-synuclein overexpressing transgenic mice with Montelukast restored memory. Montelukast treatment resulted in modulation of beclin-1 expression, a marker for autophagy, and in a reduction in the human alpha-synulcein load in the transgenic mice. Reducing the protein aggregation load in neurodegenerative diseases might be a novel model of action of Montelukast. Moreover, this work presents leukotriene signaling as a potential drug target for DLB and shows that Montelukast might be a promising drug candidate for future DLB therapy development.

**Electronic supplementary material:**

The online version of this article (10.1007/s13311-020-00836-3) contains supplementary material, which is available to authorized users.

## Introduction

Dementia with Lewy bodies (DLB) and Parkinson’s disease dementia (PDD) are age-related chronic neurodegenerative syndromes with the common features of cognitive decline, parkinsonian motor symptoms, and with an underlying pathology of alpha-synuclein aggregated in neuronal inclusions, so called Lewy bodies [1, 2]. The two syndromes are roofed under the term Lewy body dementias (LBD) [2]. DLB and PDD differ in the sequence of onset of symptoms, as in DLB, cognitive deficits preceed the parkinsonian motor symptoms, whereas in PDD, the motor symptoms arise at least 1 year before the cognitive decline. The cognitive deficits and its consequences for the patients rank as one of the key unmet needs of LBD therapy [3]. This is further underscored by the fact that LBD comprises, after Alzheimer’s disease, the second most common type of degenerative dementia in patients older than 65 years. Currently available symptomatic treatments such as cholinesterase inhibitors have limited efficacy in improving cognition and show significant side effects [4]. The efficacy of disease-modifying approaches remains to be demonstrated [5, 6].

The exact molecular and cellular basis for the cognitive impairment in LBD is only partially understood [7]. Neuronal cell death [8], which is triggered by multiple factors such as alpha-synuclein fibrils [9], reduced levels of neurogenesis [10], impaired neuronal autophagy [11] and lysosomal dysfunctions [12], and neuroinflammation [13] triggered by microglia dysfunctions [14], have been implicated in LBD. We hypothesize that therapeutic approaches addressing a spectrum of these neuropathological aspects, eventually through a pleiotropic mode of action, might have a chance of improving cognitive functions in LBD, and may be more promising than monospecific and single mode of action drugs.

One of the molecular signaling systems that is implicated in a number of pathological hallmarks of chronic neurodegenerative diseases such as LBD is leukotriene signaling (for review, see [15]). In the aged and neurodegenerative brain, cysteinyl-leukotrienes (Cys-LTs) contribute to neuroinflammation, enhance neurodegeneration, induce BBB disruption, and inhibit neurogenesis. Cys-LTs act via signaling through leukotriene receptors CysLT1R, CysLT2R, and GPR17, which are expressed in the brain, in particular on endothelial cells, microglia, neurons, and on stem and progenitor cells [16–18]. Genetic modulation of the leukotriene system has been performed in the context of Alzheimer’s disease (AD) models. There, elevated leukotriene levels exaggerate disease pathology and cognitive dysfunctions, and vice versa, blocking leukotriene signaling reduces pathology and improves cognitive functions (for review [15]). In consequence, there is increasing evidence suggesting that leukotriene receptor antagonists might be potential drug candidates to improve cognition in dementias. For example, we have recently shown that the Cys-LTR1 and GPR17 leukotriene receptor antagonist Montelukast, an approved anti-asthma drug, elevates neurogenesis, reduces neuroinflammation, restores BBB integrity, and improves learning and memory in aged rodents, which show cognitive imparments [19]. Also, in various other animal models of neurodegenerative diseases, including a model of kainic acid-induced loss of memory function, an acute Huntintgon’s disease model of quinolinic acid and malonic acid injection-induced degeneration of striatal neurons, an β-amyloid injection model of AD, a streptozotozin-induced model of cognitive decline, and in the MCAO stroke model, treatment with Montelukast attenuated behavioral deficits, which was accompanied by inhibition of neuroinflammation and reduced neuronal cell death [20–24]. Based on these data, targeting the leukotriene system might be an interesting therapeutic approach not only in Alzheimer’s disease [15], but also in various other neurodegenerative diseases including LBD.

At present, the leukotriene signaling system is completely unexplored in the field of LBD. Moreover, it is unknown if targeting leukotriene signaling might be able to alleviate symptoms associated with LBD, in particular cognitive functions. To explore that, we made use of the D-line PDGF-promoter-driven human alpha-synuclein overexpressing transgenic mouse model (alpha-syn mice) [25] and of human post-mortem brain specimen with LBD. The alpha-syn mice replicate features of human synucleinopathies such as abnormal accumulation of alpha-synuclein, development of cognitive impairments, and motor deficits. Interestingly, the cognitive deficits preceed the motor deficits in this mouse model, and therefore, the alpha-syn mice can be considered as a model for DLB [26]. We explored 5-Lox expression in the brains of alpha-syn and wild-type (WT) mice and in human DLB and age-matched control brains. Moreover, we tested the effects of a 6-week treatment with the leukotriene receptor antagonist Montelukast on neurogenesis, neuroinflammation, alpha-synuclein load, autophagy, and most importantly on cognitive functions in these mice.

## Material and Methods

### Human Brain Specimen

We used 7-μm hippocampal sections from formalin-fixed paraffin-embedded brain samples which were obtained from the Newcastle Brain Tissue Resource (NBTR) in accordance with Newcastle University ethics board and ethical approval awarded by The Joint Ethics Committee of Newcastle and North Tyneside Health Authority (reference: 08/H0906/136). Irrespective of clinical diagnoses, brains underwent neuropathological examination according to a routine protocol that uses standardized neuropathological scoring/grading systems, including neurofibrillary tangle (NFT) Braak staging [27, 28], Consortium to Establish a Registry for Alzheimer’s Disease (CERAD) scores [29], Newcastle/McKeith Criteria for Lewy body disease [30], National Institute on Aging – Alzheimer’s Association (NIA-AA) guidelines [31], and Thal phases of amyloid β deposition [32].

### Animals

Six-month-old female transgenic (TG) mice expressing human wild-type alpha-synuclein under the regulatory control of the PDGFbeta promoter (D-line) [25] and their wild-type (WT) litter mates as controls were used. Mice were housed at the central animal facility at UCSD in groups under standard conditions at a temperature of 22 °C and a 12-h light/dark cycle with ad libitum access to standard food and water. Animal care and handling were performed according to the Declaration of Helsinki and in strict accordance with good animal practice and all procedures were completed under the specifications set forth by the UCSD Institutional Animal Care and Use Committee.

### Montelukast Pharmacoexposure Analysis

Six-month-old alpha-syn and WT mice (*n* = 12) received Montelukast (10 mg kg^−1^ per day; dissolved in a 0.9% NaCl solution containing 10% ethanol) via oral gavage for seven consecutive days. One hour after the last gavage, blood was collected from every individual and serum was obtained by 1-h incubation of the blood sample at 37 °C and subsequent centrifugation at 13000 rpm. The supernatant was stored at − 20 °C until further processing. Immediately after blood collection, the animals were transcardially perfused with PBS to remove all blood from the body. Afterwards, the brain was extracted, and all tissue was stored at − 80 °C.

Mouse brains were homogenized with an equal weight of water using a Bullet Blender (Next Advance). Aliquots of homogenate from each sample were transferred to a 96 well plate and 3 volumes of acetonitrile (Fisher) containing an internal standard (200 ng/mL tolbutamide, Sigma) were added to each. After centrifugation (15 min at 1500*g*), supernatant (50 μL) was transferred to another 96-well plate and 100 μL of water added. Control brain tissue was treated in the same way to provide blank samples and calibration standards by spiking control brain homogenate with known amounts of montelukast. CSF was prepared for analysis by dilution with tolbutamide (200 ng mL^−1^) in acetonitrile; artificial CSF was used as control matrix.

Samples were analyzed using an Agilent 1290 Infinity binary pump and autosampler with detection by an Agilent 6550 iFunnel QTof mass spectrometer (MS) (Agilent) using electrospray ionization. The protonated molecules for Montelukast and tolbutamide (*m*/*z* 586.2177 and *m*/*z* 271.1116) were extracted with ± 15 ppm mass windows to generate chromatograms with suitable combinations of specificity and signal/noise. Sample aliquots (5 μL) were injected into a mobile phase initially of 98% water/0.1% formic acid (Channel A) and 2% acetonitrile/0.1% formic acid (Channel B) delivered at 0.4 mL/min to an Acquity BEH C18 50 × 2.1 mm, 1.7 μm column. The column was maintained at 50 °C in an Agilent Infinity column oven. The mobile phase composition was programmed to change linearly from 2% Channel B at 0.30 min post-injection up to 95% at 1.10 min, maintained at 95% until 1.75 min, and then returning to initial composition at 1.8 min. Column effluent was diverted to waste for the first 0.8 min post-injection to minimize source contamination. Data processing was carried out using MassHunter software (v B.05.01, Agilent UK). Calibration curves were fitted using the simplest regression model to minimize back-calculated calibration standard concentration residuals over the range of study sample concentrations.

### Montelukast Treatment

A total of 27 animals (8 TG vehicle, 8 TG Montelukast, 5 WT vehicle, 6 WT Montelukast) were treated. Montelukast sodium powder (Sigma) was first dissolved in ethanol for maximum solubility and then further diluted (1:9 ratio) with a 0.9% saline (NaCl) solution. A 3 mg/ml stock solution was prepared freshly every day and was administered daily per oral gavage (p.o.) at a dose of 10 mg kg^−1^ of body weight for 42 days. Control mice received volume-matched injections of the vehicle solution (10% ethanol in 0.9% NaCl).

### Behavioral Tests

Twenty-eight days after starting the Montelukast treatment, several standardized behavioral tests were carried out.

#### Spontaneous Locomotor Activity

Spontaneous movements of the mice were measured in a small, transparent cylinder 15.5 cm high and 12.7 cm in diameter. The cylinder was placed on a piece of glass with a mirror positioned at an angle beneath the cylinder to allow a clear view of movements along the ground and walls. Spontaneous movements were recorded for 3 min. Videotapes were viewed and rated in slow motion by an experimenter blinded to mouse genotype. The number of rears, forelimb and hindlimb steps, and time spent grooming were determined for each mouse [33].

#### Open Field

The open field locomotor test was used to determine basal activity levels of study subjects (total move time) during a 15-min session. Spontaneous activity in an open field (25.5 × 25.5 cm) was monitored for 15 min using an automated system (Truscan system for mice; Coulbourn Instruments). Animals were tested within the first 2–4 h of the dark cycle after being habituated to the testing room for 15 min. The open field was illuminated with an anglepoise lamp equipped with a 25 W red bulb. Time spent in motion was automatically collected in 3 × 5 min time bins using TruScan software. Data were analyzed for both the entire 15-min session and for each of the 5-min time blocks.

Spontaneous locomotor activity and open field parameters were assessed using photocell emitters and receptors equally spaced along the perimeter of the chamber, which create an x-y grid of invisible infrared beams. Animals being placed in the chamber move and cause beam breaks. Vertical sensors are also present to assess vertical activity levels (i.e., rearing behavior).

#### Fecal Motility

Following the locomotor activity testing, fecal pellets were collected from each mouse. The number of fecal pellets per mouse was used as readout for fecal motility.

#### Morris Water Maze

On days 35–41 after the first Montelukast administration, the water maze procedure was used to assess the ability of spatial learning and memory. The apparatus consisted of a circular swimming pool built of black plastic (180-cm diameter, 76-cm height), filled with 21 °C ± 1 °C tempered water. The tank was virtually divided into four equal quadrants, with a submerged hidden 10 × 10 cm fiberglass platform placed 3 cm below the water surface in the middle of the target quadrant. The position of the platform was kept unaltered throughout the learning sessions. In the testing room, several big black cue symbols were put on each wallfor spatial orientation. The water maze task was carried out twice a day for five consecutive days. One day before starting the learning experiment (day 0), each mouse was put into the water and was allowed to locate the submerged platform for 60 s. If the animal failed to find the platform within the 60 s, it was guided onto the platform and allowed to remain there for 10 s. For the learning tasks on days 1–5, each mouse was put into the water at one of four starting positions, the sequence of which being selected randomly. In each trial, a ceiling time of 60 s to find the platform was defined. The escape latency time to locate the hidden platform and the distance moved during the trial were recorded with the camera software as indices of spatial learning. On the final day of testing (day 8), after the learning phase of the experiment, a probe trial was performed. Here, the platform was removed and each animal was allowed to explore the pool for 60 s. The time spent in the former platform zone (“correct zone”) was recorded.

### Perfusion and Tissue Processing

On day 42, mice were deeply anesthetized using a ketamine (20.38 mg/ml), xylazine (5.38 mg/ml), and acepromazine (0.29 mg/ml) mixture. Transcardial perfusion was performed with 0.9% NaCl solution, followed by a 4% paraformaldehyde, 0.1 M sodium phosphate solution (pH 7.4). The brains were dissected and post-fixed for 4 days in a 4% paraformaldehyde solution at 4 °C. Brains were cut using a sliding microtom into 40-μm saggital sections on dry ice and then cryoprotected in 30% sucrose, 0.1 M sodium phosphate solution (pH 7.4) and kept at − 20 °C.

### Immunohistochemistry

Immunohistochemistry (IHC) of mouse tissue was performed on free-floating sections as previously described [19]. Antigen retrieval was performed depending on the used primary antibody by heating the sections for 15–20 min in 1x citrate buffer (pH = 6.0, Sigma).

#### Diaminobenzidine Staining

The following primary antibodies were used for chromogenic IHC: rabbit anti-Doublecortin (1:300, Cell Signaling), goat anti-Doublecortin (1:350, Santa Cruz), ms anti-5-LOX (1:100, BD Transduction Laboratories), rabbit anti-Iba1 (1:300, Wako), goat anti-PCNA (1:250, Santa Cruz). Secondary antibodies: donkey anti-mouse, anti-goat, anti-rabbit, anti-rat biotinylated (all 1:500, Jackson Immuno Research). After incubation in secondary antibodies, sections were incubated in Avidin-Biotin-Peroxidase solution (Vectastain ABC Kit) and visualization was performed using the diaminobenzidine (DAB) peroxidase substrate kit (SK-4100, Vector Laboratories). All quantification of immunohistochemistry was done blinded (i.e., the experimenter was not aware of the group to which the analyzed material belonged). Every tenth section (40-μm interval) of one hemisphere was selected from each animal and processed for immunohistochemistry. Cells positive for the respective marker (PCNA, DCX, or Iba1) within the dentate gyrus were counted on a Zeiss Axioplan microscope. To further analyze Iba1+ cells, their soma size was measured in the GL and SGZ in 2 images per mouse taken with a 20× objective. First, the cell somas were traced manually in the GNU Image Manipulation Program (GNU GIMP 2.6.11, www.gimp.org). Afterwards, the size of the somas was measured with the “Analyze Particles” function of the software ImageJ (ImageJ 1.45s, National Institutes of Health, https://imagej.nih.gov/ij).

#### Fluorescence Staining

The following antibodies were used for fluorescence IHC: rat anti alpha-synuclein (1:50, Enzo), mouse anti aggregated alpha-synuclein clone 5G4 (1:500, Merck), rabbit anti NeuN (1:200, Cell Signaling), rabbit anti Beclin-1 (1:500, Sigma), rabbit anti Lamp2A (1:200, Abcam), guinea pig anti NeuN (1:500, Merck), rabbit anti TSPO (1:250, Novus Biologicals), goat anti-Iba1 (1:500, Abcam), ms anti-5-LOX (1:50, BD Biosciences). For alpha-synuclein staining, antibody incubation time was increased from 1× overnight to a total of 5 days on 4 °C. Sections were extensively washed in PBS and incubated for 3 h at RT in secondary antibodies all diluted 1:1000. The following secondary antibodies were used: donkey anti-rabbit Alexa Fluor 568 or Alexa Fluor 647, donkey anti-goat Alexa Fluor 568, donkey anti-rat Alexa Fluor 488 or Alexa Fluor 568 (all Invitrogen/Molecular Probes), donkey anti-guinea pig, and donkey anti-mouse Alexa Fluor 647 (Jackson Immuno Research). Nucleus counterstaining was performed with 4′,6′-diamidino-2-phenylindoledihydrochloride hydrate (DAPI 1 mg/mL, 1:2000, Sigma). Sections were extensively washed in PBS and mounted onto microscope glass slides (Superfrost Plus, Thermo Scientific). Brain sections were cover slipped semi-dry in ProLong Gold Antifade Mountant (Life technologies) or Fluorescence Mounting Medium (Dako). A confocal scanning laser microscope (Zeiss LSM 700, Germany) equipped with the Zeiss Axio Vision imaging system, with the LSM software (ZEN black 2011), was used for imaging of brain sections labeled with fluorescent antibodies. TSPO/Iba1 staining: Four visual fields (400 × 400 × 40 μm) of dentate gyrus in 40× magnification were taken per animal. Percentage area of TSPO in the dentate gyrus was measured with the help of ImageJ.

##### a-Syn/NeuN and 5-Lox staining

Each slide contained sections of a representative 10th of mouse hemisphere. Three different sections containing triangle shaped dentate gyrus were selected from each slide and z-stacks of two visual fields (400 × 400 × 40 μm) in 20× magnification were taken in order to photograph the whole dentate gyrus per section. Using ImageJ, z-stack was merged to maximum projection and region of interest (ROI), containing dentate gyrus plus hilus, was defined based on NeuN staining, or DAPI for analysis of 5-Lox, respectively. Percentage area was measured in ROI, also using ImageJ software. Additionally, a-Syn + cells were counted in the same ROI. Thickness of DG was measured based on Neun staining. Beclin1/NeuN and Lamp2A/NeuN staining: ImageJ was used to identify the percentage area of each autophagy-lysosomal pathway (ALP) marker, the autofluorescence signal and the colocalization of both. The selected ROI included only neurons of the granula layer of the dentate gyrus. For each Beclin-I stained brain slice, a z-stack of 5 slices was recorded and merged for analysis. For the Lamp2a staining only the one most intensely stained picture of the z-stack was used for analysis. To select valid, specific immunostaining signals for every antibody, the threshold for detection was adjusted individually on each merged picture. Control brain slices were stained with secondary antibodies only, to confirm specificity of the ALP marker staining. The autofluorescence, recorded at 568 nm wavelength, was most likely caused by age associated lipofuscin accumulations. To avoid recording false positive ALP marker expression, the autofluorescence co-staining signal was subtracted from the ALP marker channel by using ImageJ.

#### FFPE Immunohistochemistry

Human samples were immunohistologically stained as follows: formalin-fixed paraffin-embedded (FFPE) human tissue sections were deparaffinized by incubation in Roti®-Histol (Roth-Lactan), rehydrated by a graded series of ethanol and rinsed in distilled water for 5 min. For antigen retrieval, the slides were heated in 0.01 M sodium citrate buffer, pH 6.0 at 100 °C for 25 min, followed by 3 washes in PBS + 0.1% Tween. Endogenous peroxidases were quenched with 0.3% hydrogen peroxide for 20 min. Sections were blocked with a solution composed of PBS, 0.1% TWEEN 20 (Sigma), 1% bovine serum albumin, and 0.2% teleostean gelatin (Sigma) for 20 min, and incubated overnight at 4 °C with a mouse anti 5-LOX antibody (1:50; 610695, BD Pharmingen) diluted in the blocking solution. After incubation, the sections were washed in PBST, incubated with a biotinylated donkey anti-mouse antibody (1:500, Jackson Immuno Research) for 1 h, washed in PBST, and incubated for 1 h in a peroxidase-avidin complex solution (Vectastain Elite ABC kit; Vector Laboratories). The peroxidase activity of immune complexes was revealed with a solution of PBS containing 0.25 mg/mL 3,3-diaminobenzidine (Vector Laboratories), 0.01% H2O2, and 0.04% NiCl2. Hematoxylin (RE7107-CE, Leica) was used for cell nuclei counter stain. Sections were dehydrated by a graded series of ethanol and mounted with Neo-Mount (Merck). VS120 Virtual-Slide-Mikroskop, 20× objective (Olympus) was used for qualitative pictures of human tissue section.

#### Statistical Analysis

Statistical analysis was performed using the GraphPad Prism 7.0 software (GraphPad Software). Normality distribution of continuous variables was assessed by Shapiro-Wilk test. Grubb’s test (*p* < 0.05) of QuickCals GraphPad Sotfware (http://graphpad.com/quickcalcs/grubbs1) was used to identify statistical outliers. Comparing two groups, the unpaired Student’s *t* test was used. For comparing multiple groups, one-way ANOVA was used followed by Tukey’s multiple comparison test. For analysis of learning behavior over time, two-way ANOVA followed by Sidak post hoc test was used. Correlation analyses were performed using Pearson’s correlation coefficient. *p* values of *p* < 0.001 (***) were considered most significant, *p* < 0.01 (**) highly significant, and *p* < 0.05 (*) significant. All values were expressed as means ± standard deviation (SD), unless otherwise stated.

## Results

### Increased 5-Lox Expression in DLB Brains

**Fig. 1:**
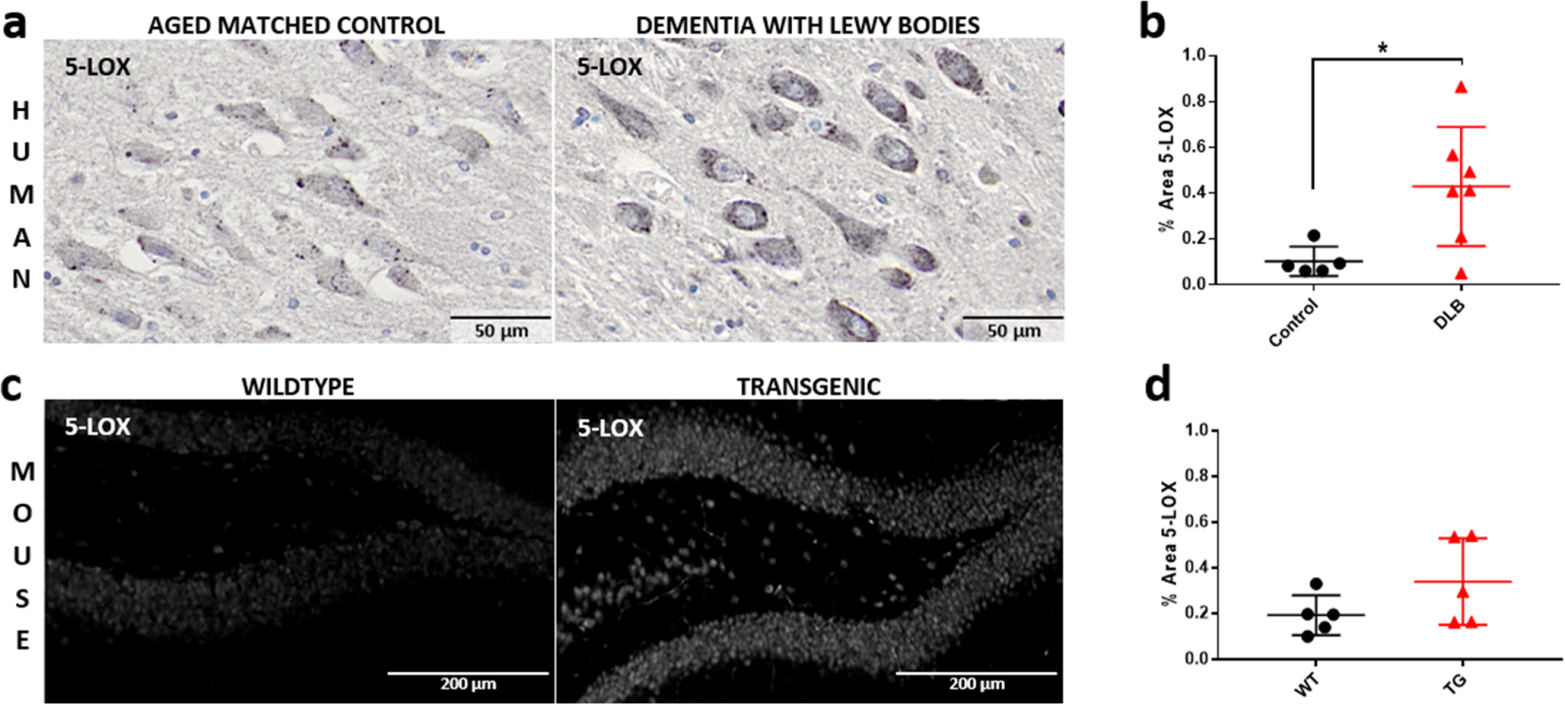
5-Lox expression is upregulated in the hippocampus of DLB brains. (**a**) + (**b**) DAB-5-Lox immunoreactivity in hippocampal neurons in post-mortem brains of DLB patients compared to aged controls. (**a**) The images show representative 5-Lox immunoreactivity of a 75-year-old DLB patient and a 90-year-old healthy control. Note the stronger 5-Lox immunoreactivity in the DLB brain compared to the control brain. (**b**) 5-Lox DAB staining of 5–7 brains per group, one section each, were quantitatively analyzed. Brain sections of patients with diagnosed DLB showed significantly higher amount of 5-Lox than controls. (**c**) Representative image of immunofluorescence 5-Lox staining in dentate gyrus hippocampal neurons of 6-month-old alpha-syn and WT control mice. Note the intensive 5-Lox immunoreactivity in the alpha-syn mice compared to weak staining in the WT littermate controls. (**d**) Quantitative analysis of 5-Lox staining in 6-month-old mice, transgenic animals *versus* wild-type (*n* = 5). Data are shown as mean ± SD. *p < 0.05, **p < 0.01, ***p < 0.001. Unpaired Student’s *t* test was performed (**b**, **d**). Scale bars: (**a**) 50 μm, (**c**) 200 μm

5-Lox is the key enzyme in the synthesis of leukotrienes and its expression is indicative for leukotriene production. Here, we analyzed 5-Lox immunoreactivity in post-mortem brains with DLB and in brains of 6 months old transgenic alpha-syn mice expressing human wild-type alpha-synuclein under the regulatory control of the PDGFbeta promoter [25] and wild-type (WT) litter mates. At this age, the TG animals have already cognitive deficits but do not suffer yet of motor impairments [26]. In the human DLB brain, more specifically in the hippocampus, we noticed a strong 5-Lox immunoreactivity, although only a faint signal was present in the age-matched control (Fig. 1a). This was systematically and quantitatively assessed in a set of 7 DLB brains and 5 healthy control brains (Table 1). The analysis revealed a significantly higher level of 5-Lox immunoreactivity in the DLB brains (Fig. 1b). Similarly, we detected in some animals a stronger 5-Lox immunoreactivity in the hippocampus in the alpha-syn mice compared to WT mice (Fig. 1c). This, however, was quite variable and did not reach significance in 5 animals per group. Nevertheless, as the human data are quite convincing, we suggest that leukotriene synthesis migh be elevated in DLB brains.

**Table 1:** Neuropathology of DLB cases

Pathologic diagnosis	McKeith	Age at death	Gender
DLB	Neocortical	81	Male
DLB	Neocortical	81	Male
DLB	Neocortical	83	Male
DLB	Neocortical	75	Female
DLB	Neocortical	79	Female
DLB	Neocortical	81	Female
DLB	Neocortical	91	Female
Nothing abnormal beyond age	No LBD	70	Male
Nothing abnormal beyond age	No LBD	73	Male
Nothing abnormal beyond age	No LBD	73	Male
Nothing abnormal beyond age	No LBD	90	Female
Nothing abnormal beyond age	No LBD	93	Female

Pathological diagnosis, age, gender, and McKeith Criteria for Lewy body disease of hippocampal sections from formalin-fixed paraffin-embedded brain samples which were obtained from the Newcastle Brain Tissue Resource (NBTR) in accordance with Newcastle University ethics board and ethical approval awarded by The Joint Ethics Committee of Newcastle and North Tyneside Health Authority (reference: 08/H0906/136)

### Montelukast Treatment Improves Memory in Alpha-Syn Mice

**Fig. 2:**
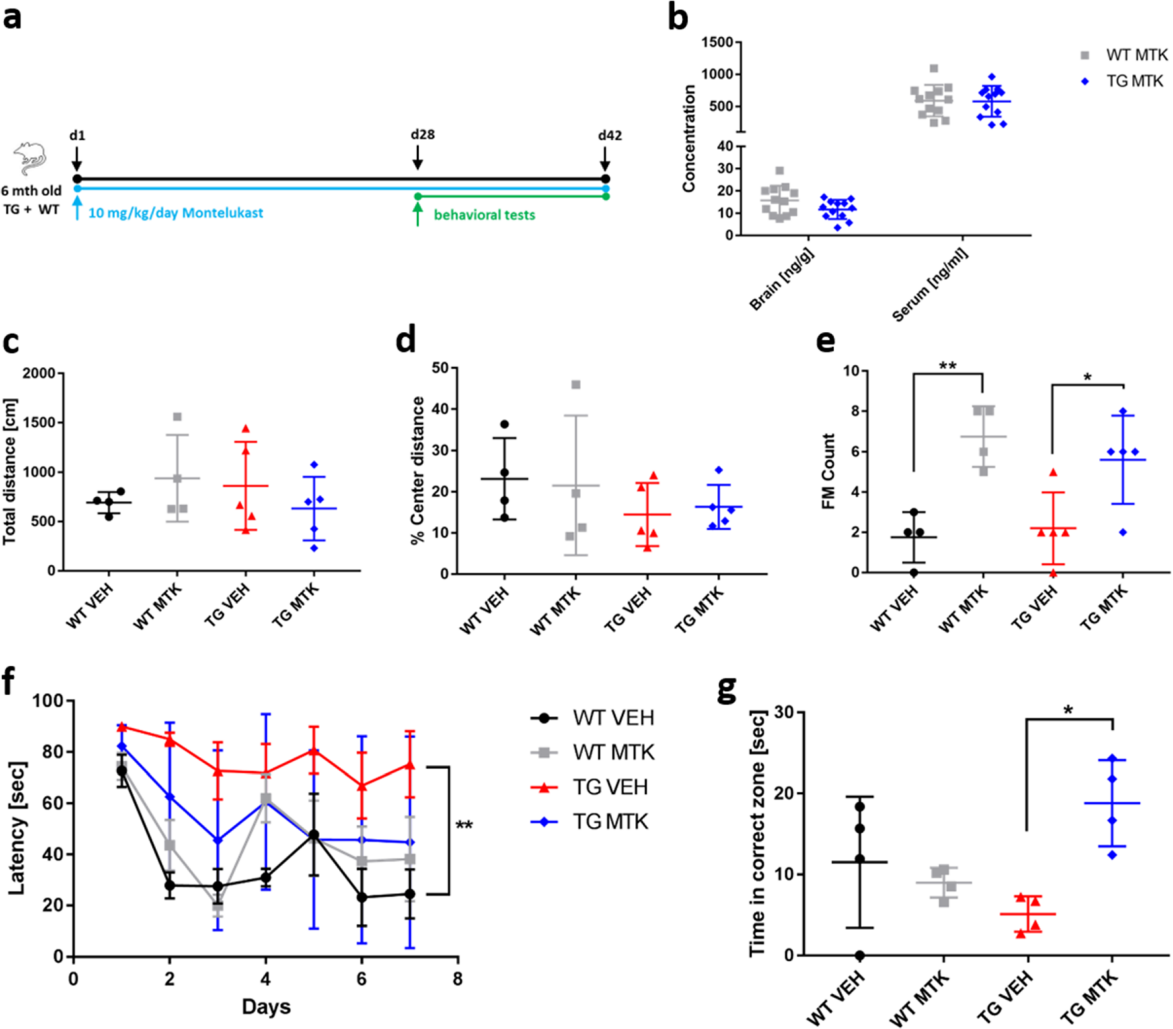
Montelukast treatment improves memory in alpha-syn mice. (**a**) Experimental design. (**b**) Pharmacoexposure data: 6-month-old alpha-syn mice received daily 10 mg kg^−1^ Montelukast via oral gavage for seven consecutive days. One hour after the last gavage, blood was drawn, brains were removed, and levels of Montelukast were quantified. Montelukast levels were in the expected range of approximately 500 to 1000 ng/ml in the serum and 10–20 ng/ml in the brain of WT and of TG animals. (**c**–**g**) Behavioral analyses. (**c**–**e**) open field: (**c**) total distance, alpha-syn animals did not differ from WT animals, and Montelukast treatment did not affect animals in these parameters. (**d**) % center distance: TG animals tended to spend less time in the center zone of the arena and more time in the periphery. Montelukast treatment did neither affect WT nor TG animals in this parameter. (**e**) Fecal motility (FM), i.e., the number of feces, demonstrates that the TG animals did not differ from WT animals, but Montelukast treatment significantly increased the fecal motility in both genotypes. (**f**, **g**) learning and memory were analyzed in the Morris water maze: (**f**) Latency times to find the hidden platform. Although vehicle treated WT animals showed a steep learning curve, TG animals failed to learn the location of the platform. Transgenic animals learned significantly worse than wildtype animals. In contrast, Montelukast treated TG animals showed a similar learning performance compared to the WT animals. (**g**) Montelukast treated TG animals significantly performed better than the vehicle treated animals and reached a level of performance similar to the WT animals. Data are shown as mean ± SD. *p < 0.05, **p < 0.01, ***p < 0.001. One-way ANOVA followed by Tukey’s post hoc test (**c**–**e**, **g**), and two-way ANOVA with Sidak post hoc tests (**f**). N 4–5 per group

We had previously shown that a 6-week treatment with Montelukast improved learning and memory in aged rats [19]. Here, we made use of this treatment regimen to test the effects of Montelukast on structural and functional outcomes in the alpha-syn TG and WT animals. For that, 6 months old TG mice and their WT litter mate controls were treated daily with 10 mg/kg Montelukast by oral gavage for a total period of 6 weeks. During the last 2 weeks of treatment, behavioral assessments, i.e., spontaneous locomotor activity in an activity cage and in the open field, autonoumous nervous system function indicated by fecal motility, and cognitive function in the Morris water maze test were performed. At the end of the treatment period, animals were perfused and brains were prepared for immunohistological analyses (Fig. 2a). In a second cohort of drug-treated animals, serum and brain tissue was analyzed for the levels of Montelukast in the central nervous system of TG and WT mice and to provide pharmacoexposure data.

As expected, we found high levels of Montelukast in the range of 500 to 1000 ng/ml in the serum of WT and of TG animals (Fig. 2b). Further, and in accordance with findings from our previous work in rats [19], the levels of Montelukast in the brain were much lower, approximately 10–20 ng/ml, which however is in the range of the Montelukast IC50 and therefore in the pharmacological active range [19]. Pharmacoexposure in WT and in TG animals were comparable suggesting that the bioavailability of Montelukast in the TG animals was not disturbed.

In a first set of behavioral tests, we assessed spontaneous locomotor function and the effects of the Montelukast treatment on this activity by analyzing the number of beam breaks in a home cage. The 7.5 months old TG animals did not differ from WT animals and Montelukast did not affect this parameter (Supplementary Figure 1). This confirms that the TG animals do not have a noticeable motor deficit at this age, and moreover, that Montelukast treatment does not induce any motor adverse events. In the open field, the TG animals tended to spend less time in the center zone of the arena and more time in the periphery suggesting that the TG animals might suffer from a higher level of anxiety. This, however, did not reach significance. Montelukast treatment did neither affect WT nor TG animals in this parameter (Fig. 2c, d).

We analyzed fecal motility, i.e., the number of feces, as an indicator of bowel activity and autonomic nervous system function, as reduced bowel activity is known to be one of the symptoms in LBD [1]. The TG animals did not differ from WT animals in fecal motility, but interestingly, Montelukast treatment significantly increased the fecal motility in both genotypes, i.e., WT and TG (Fig. 2e). Whether this is related to a direct action of Montelukast on the autonomic nervous system or an unspecific side effect of Montelukast is unclear. Irrespectively, it can be interpreted as a beneficial effect.

Finally, we assessed spatial learning and memory in the Morris water maze. Compared to the vehicle treated WT animals, which showed strong learning already within the first 2 days of the experiment, the TG animals failed to learn the location of the platform during the 7 days of the test. The Montelukast treated alpha-syn animals showed a tendency for increased learning compared to the vehicle treated ones, however, the improvement did not reach statistical significance (Fig. 2f). The Montelukast treated TG animals had nevertheless a similar performance compared to the WT animals supporting the efficacy of Montelukast to improve learning in this animal model. On day 8, 1 day after the learning acquisition, animals were tested in the probe trial for their capacity to remember the location of the platform by analyzing the time the animals spent in the zone where the platform used to be. Here, the Montelukast treated TG animals performed remarkably better than the vehicle treated animals and reached a level of performance similar to the WT animals. In summary, we confirm that the TG animals at the age of 7 to 8 months do not have any obvious motor deficits, but have severe cognitive deficits. Montelukast did not induce any side effects neither in WT nor in TG animals, but restored memory function in the TG mice.

### Montelukast Treatment and Microglia in the Brains of the DLB Animal Model

Based on our previous work, which showed neurogenesis promoting effects of Montelukast in aged rats [19], we first analyzed proliferating and doublecortin positive neural stem and progenitor cells in the hippocampus of TG and WT animals and assessed the effects of Montelukast treatment on neurogenesis. In contrast to a previous publication [34], the alpha-syn TG mice did not show impaired hippocampal neurogenesis, i.e., numbers of proliferating and of doublecortin positive cells, in the dentate gyrus subgranular/granular layer (data not shown). A possible explanation for these differences is that these animals have been bred into a genetic background different from the initial one. Further, Monteluakst treatment had no effects on these neurogenesis parameters, neither in WT nor in the alpha-syn animals (data not shown).

**Fig. 3:**
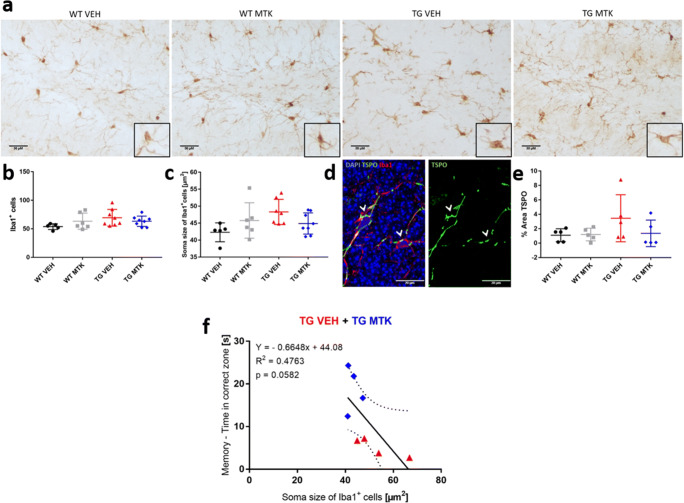
Montelukast treatment and effects on microglia. (**a**) Iba1 immunostaining in the dentate gyrus of WT and alpha-syn mice treated with vehicle or Montelukast. Insert show representative cellular morphology. Control stainings, in which the primary goat Iba1 antibody was omitted, excluded unspecific labelling of the secondary antibody. (**b**) Number, and (**c**) cell soma size of Iba1 positive cells of the different genotype and treatment groups. (**d**) TSPO/Iba1 double immunofluorescence staining illustrating localization of TSPO primarily in Iba1 cells. (**e**) Quantitative analysis of TSPO staining intensity. (**f**) Correlation microglia soma size with memory. Correlations were performed by Pearson correlation. Data are shown as mean ± SD. *p < 0.05, **p < 0.01, ***p < 0.001. One-way ANOVA was performed (**b**, **c**, **e**). Scale bars: (**a**) 30 μm, (**d**) 20 μm

Next, we analyzed microglia as an indicator of neuroinflammation. First, we assessed the number of Iba1 positive microglia in the hippocampus. Montelukast had no significant effects on this parameter (Fig. 3a, b). Microglia soma size is used as morphological indicator for microglia activation [35] and has been suggested to correlate with dystrophic and dysfunctional microglia [36]. Interestingly, bigger soma size is a hallmark of microglia in aging and neurodegenerative diseases [36]. Iba1 positive microglia in the TG animals exhibited a trend towards a larger soma size compared to WT animals, and Montelukast treatment slightly but not significantly reduced microglia soma size (Fig. 3c). To further investigate a potential effect of Montelukast on microglia activity, we analyzed TSPO immunoreactivity in the hippocampus. TSPO, a benzodiazepine receptor (PBR) present on the mitochondria of activated microglia, has been shown to be upregulated under neuroinflammatory conditions. Further, the TSPO ligand PK11195 is used as a PET tracer to image microglia and neuroinflammation in patients with neurodegenerative disease, including patients with Lewy body pathologies [37]. TSPO immunoreactivity did, as expected, co-localize with Iba1 postive microglia (Fig. 3d). In the TG animals, TSPO immunoreactivity was slightly higher compared to WT animals, but overall, there were no significant differences between the groups (Fig. 3e). In summary, microglia in alpha-syn TG animals showed some minor differences compared to WT animals, and Montelukast had only minor effects on the microglial in this animal model. The microglia soma size did slighty but not significantly (*p* = 0.0582) correlate with memory function, i.e., the bigger the microglia cell body, the lower the performance of the animals to memorize (Fig. 3f).

### Montelukast Treatment Reduced Alpha-Synuclein Load in the DLB Animals

**Fig. 4:**
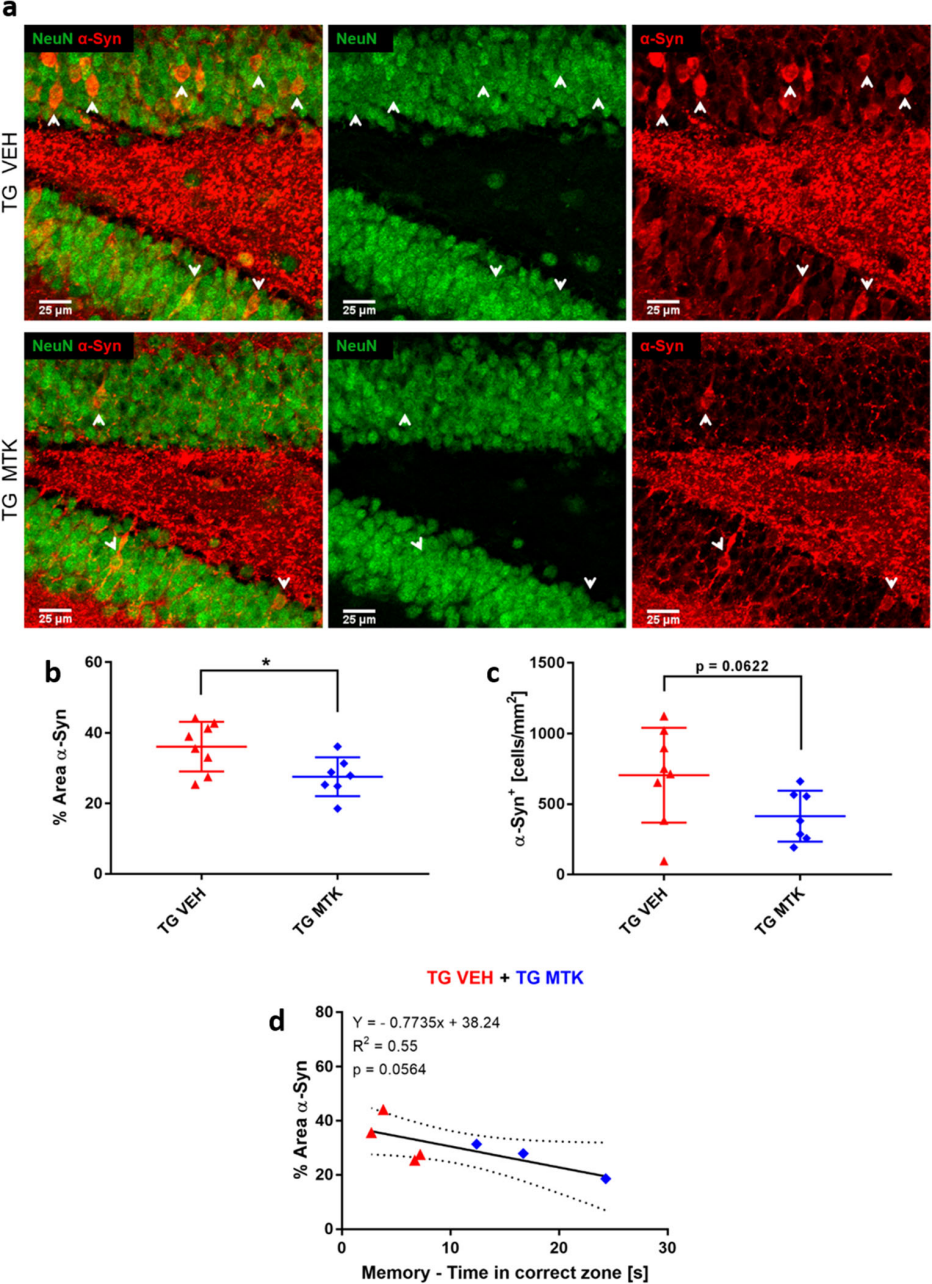
Montelukast reduces alpha-synuclein in alpha-syn mice. (**a**) Human alpha-synuclein (red) and NeuN (green) immunostainings in TG mice treated with vehicle or Montelukast. Note the strong human alpha-synuclein positive accumulation in the granular cell layer of the dentate gyrus of the vehicle treated TG animals, which was hardly detected in the Montelukast treated animals. (**b**) Quantitative analysis of the percentage area of the staining confirmed the lower levels of alpha-syn in the Montelukast treated group. (**c**) Number of human alpha-syn positive neuronal cell bodies in the dentate gyrus granular cell layer. (**d**) Correlation %Area aSyn with memory. Correlation was performed by Pearson correlation. Data are shown as mean ± SD. *p < 0.05, **p < 0.01, ***p < 0.001. Unpaired Student’s *t* test was performed (**b**, **c**). Scale bars, 25 μm

Because alpha-synuclein aggregation is the typical histopathological hallmark of LBD and presented also in the alpha-syn TG animals [25], we investigated a putative effect of Montelukast on the alpha-synuclein load. Strong human alpha-synuclein positive neurons were visible in the granular cell layer of the dentate gyrus of the TG animals (Fig. 4a). In Montelukast treated TG animals, the alpha-synuclein staining (percentage area) in the granule cells was significantly less intense compared to the vehicle treated group (Fig. 4a, b). The number of granule cells showing the high human alpha-synuclein load was also slightly reduced in the Montelukast treated TG animals, although this did not reach significance (*p* = 0.0622) (Fig. 4c). The reduced alpha-syn intensity showed a strong trend for correlation with memory function (*p* = 0.0564) (Fig. 4d). Next, we used an antibody that specifically detects the aggregated form of alpha-syn. Here, we did not observe any difference in aggregated alpha-syn load between the Montelukast and vehicle treated animals (data not shown).

**Fig. 5:**
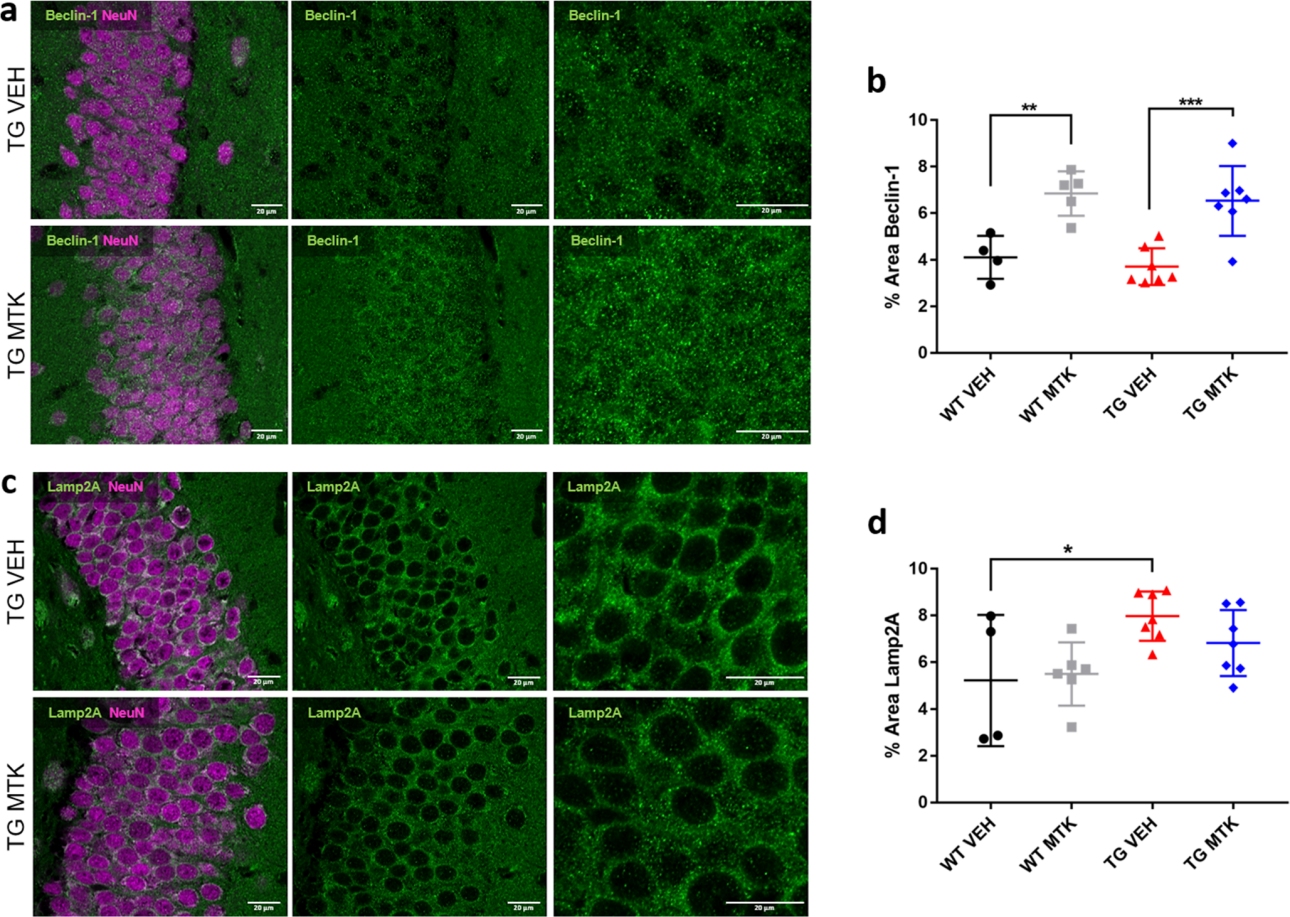
Montelukast affects autophagy markers in alpha-syn mice. (**a**) Immunofluorescence of Beclin-1 (green) and NeuN (violet) in the granular cell layer of the dentate gyrus/hippocampus in alpha-syn transgenic mice treated with vehicle or Montelukast. Note the more intense staining in the Montelukast treated group. (**b**) Quantitative analysis of the Beclin-1 staining intensity. (**c**) Immunofluorescence images of Lamp2A (green) and NeuN (violet) in the granular cell layer of the dentate gyrus/hippocampus in alpha-syn transgenic mice treated with vehicle or Montelukast. (**d**) Quantitative analysis of the Lamp2A staining intensity. Data are shown as mean ± SD. *p < 0.05, **p < 0.01, ***p < 0.001. One-way ANOVA followed by Tukey’s post hoc test was performed. Scale bars, 20 μm

The mode of action of the Montelukast induced reduction in the alpha-synuclein load in the TG animals is unclear at present, but it might involve a Montelukast induced selective death of the alpha-syuclein positive granule cells. To address this possibility, we first analyzed the thickness of the dentate gyrus granule cell layer in the TG animals. This parameter did not differ in Montelukast and vehicle treated TG animals, and therefore, it is unlikely that Montelukast induced a selective cell death of these neurons (Supplement Figure 2). Another possibility is that Montelukast treatment promotes autophagy. This might be of high relevance, because impaired autophagy is central to many neurodegenerative diseases, in particular in protein aggregation diseases, in which impaired autophagic clearance contributes to the accumulation of protein aggregates [38]. The two autophagy pathways that have been implicated in synucleopathies are macroautophagy and chaperone-mediated autophagy, which require Beclin-1 in the case of macroautophagy and LAMP2a in the case of chaperone-mediated autophagy [39, 40]. To test whether Montelukast treatment induces changes in autophagy, we analyzed Beclin-1 and LAMP2a using immunohistochemistry. Beclin-1 immunoreactivity was similar in TG and WT animals, and the Montelukast treatment significantly elevated the Beclin-1 expression in both genotyes (Fig. 5a, b). In contrast to that, LAMP2a levels were higher in the TG compared to WT animals, and Montelukast treatment did not have an effect on this parameter (Fig. 5c, d). This suggests that Montelukast might improve macroautophagy but not the chaperone-mediated autophagy pathway.

## Discussion

With the present work, we demonstrate for the first time that the leukotriene receptor antagonist and anti-asthma medication Montelukast improves memory in a synucleopathy animal model of dementia, i.e., the human alpha-synuclein over-expressing mice. The underyling scientific rational is based on the finding that expression of 5-Lox is elevated in DLB brains suggesting that also leukotriene levels might be elevated in such brains. This is most likely not specific for DLB and might play a role also in other alpha-synucleopathies. The leukotriene receptors have been localized in the brains of MPTP mice, a widely used acute model of dopaminergic loss [41]. CysLTR1, CysLTR2, and GPR17 are expressed in TH-positive dopaminergic neurons and microglia, whereas CysLTR2 expression was also found in astrocytes and was increased after MPTP-induced damage [41]. Also, leukotrienes contribute to vulnerability of dopaminergic neurons in response to neurotoxicity, and leukotriene inhibition prevents MPTP-induced death of dopaminergic neurons [41–43]. Leukotriene receptor inhibition through Montelukast has been shown to protect dopaminergic neurons against 6-OHDA-induced neurotoxicity and its administration significantly attenuated the production of neurotoxic cytokines such as TNFalpha and IL-1beta from activated microglia in the substantia nigra and striatum following 6-OHDA treatment [44]. Therefore, Montelukast might be a potential drug candidate not only in DLB but also in other synucleophaties. This is further supported by a recent study demonstrating that salbutamol, a brain-penetrant asthma medication, is associated with reduced risk of developing Parkinson’s disease [45].

Besides in synucleopathy-related disorders, the leukotriene system might be a relevant drug target in various other neurodegenerative diseases such as AD (for review see [15]), in acute CNS lesions such as stroke [24] and spinal cord injury [46, 47], and in a variety of other non-CNS chronic diseases [48]. For example, overexpression of ALOX5, the gene encoding 5-Lox resulted in increased amyloid β plaque formation, increased levels of γ-secretases and increased levels of total tau and phosphorylated tau in the 3xTG mouse model of AD [49]. Vice versa, an ALOX5 knock-out mediated deficiency of 5-Lox reduced the levels of amyloid beta and its depositions in the brain of Tg2576 AD mice [50]. Similarly, deletion of ALOX5, the gene encoding 5-Lox, leads to memory improvement and enhanced synaptic integrity, and to a reduction in amyloid beta and tau pathology in transgenic AD mice [51]. Besides genetic approaches, pharmacologic targeting of leukotriene synthesis and of leukotriene receptors has been shown to modulate disease pathology and improve cognitive functions in animal models of AD and in aged wild-type rodents. For example, Zileuton, a specific 5-Lox inhibitor, reduced amyloid beta levels and plaque deposition and the levels of insoluble and of hyperphosphorylated tau, and stabilized or improved cognitive function in animal models of Alzheimer’s disease [52–54]. In the MCAO model of stroke, leukotriene receptor blockade through Montelukast reduced lesion size progression, improved fiber connectivity and functional recovery [24].

What is the mode of action of leukotriene or leukotriene receptor antagonization? As recently reviewed [15], leukotriene signaling can contribute to neuroinflammation, neurodegeneration, BBB disruption, and to impairment in neurogenesis. Although in aged rats the Montelukast induced learning and memory improvements correlated with increased neurogenesis [19], we did not observe any neurogenesis boosting effects by Montelukast in the alpha-syn TG animals nor in the WT animals. This, however, might be due to the fact that i) neurogenesis was not affected in the TG mice compared to WT animals, and ii) animals, also WT, were of relatively young age (6–7.5 months), in which neurogenesis has not yet declined [55]. Thus, Montelukast improved cognition in the alphy-syn animals most likely in a neurogenesis independent mode of action. As in our recent aging study [19], we did observe moderate, although not significant, Montelukast treatment-induced changes in microglia. This might be of relevance because a broad body of evidence highlighted that neuroinflammation is central in alpha-synucleopathies such as LBD (for review see [13]). Moreover, in synucleopathies, like in many other neurodegenerative diseases, microglia are the brain’s most relevant resident innate immune cells [14]. Nevertheless, the possible effects of Montelukast on neuroinflammation and on microglia certainly require further in-depth analysis, considering also the very recent developments on gene expression profiling of microglia phenotypes at the single cell level [56, 57]. The latter has underscored the heterogeneity of microglia in the brain and identified a signature for degeneration associated microglia. Further, Montelukast might, and we cannot exclude this at the moment, exert its effects on the CNS and on neuroinflammation via modulating the peripheral immune system. Indeed, there is ample evidence for anti-inflammatory effects of Montelukast in peripheral tissues and organs. Montelukast treatment reduces production of IL-6, IL-8, GM-CSF, INF-gamma, RANTES, TNF-alpha, CRP, and CCL-2 [58–68] and elevates the levels of the anti-inflammatory cytokine IL-10 [69]. Of importance, inflammatory cytokines and inflammation in serum correlate with disease progression of PD and PDD. PD patients have elevated levels of CRP, CCL-2, IL-6, and of TNF-alpha, correlating partially with fatigue, depression and anxiety, and with cognitive deficits [70–72]. Moreover, the levels of cytokines and chemokines (CCL-2, RANTES, MIP-1alpha, IL-8, IFNgamma, IL-1beta and TNFalpha) produced by peripheral blood mononuclear cells (PBMCs) derived from PD patients are significantly elevated [73]. Obviously, in cases where Monteluakst might reduce neuroinflammation, an optimal timing of treamtnet might be crucial. For example, early treatment of neurodegenerative diseases might potentiate the effects of Montelukast. This has been suggested in a Norwegian retrospective analysis of a drug prescription database, the Norwegian Prescription Database (NorPD), in which people elder than 50 that have been prescribed Montelukast were compared to people prescribed inhalative corticosteroids. The conclusion was that the Montelukast group had a significant lower probability of requiring a dementia drug later in life and of requiring to be placed in a nursing home [74]. Another aspect might be the potential of Monteluakst to affect, besides cognition and memory, other non-motor sympotms of synucleopathies such as depression [75]. The latter is generally associated with neuroinflammation [76]. Also, of note, there is a strong comorbidity between DLB and depression [77].

An unexpected and novel finding of the present work was that Montelukast treatment induced reduction in the alpha-synulcin load in the dentate gyrus. The analysis of autophagy markers suggests that Montelukast might restore autophagy in the alpha-syn mice. The underlying mode of action is unknown, as the effects of leukotrienes and of leukotriene inhibition on autophagy are rather unexplored [78, 79]. Nervertheless, this might be of huge importance, as in synucleopathies as well as in other neurodegenerative diseases autophagy is typically impaired, and restoration of autophagy is discussed as a potential therapeutic strategy. There is a strong body of evidence that in synucleinopathies the clearance of toxic synuclein is impaired, contributing to neurodegeneration (for review, see [80]). In PD and LBD mouse models and transgenic cell lines, dysfunctional lysosomes were shown to impair the autophagy process, which resulted in autophagosome accumulations in neurons [80]. The putative effect of Montelukast on autophagy certainly requires further investigations. A higher expression of beclin-1 might indicate an improved macroautophagy. Also, the unchanged levels of Lamp2a suggest that chaperone-medicated autophagy is not alterend. However, because there is a strong crosstalk between these two forms of autophagy, in particular in neurodegenerative diseases [81], we cannot really differentiate between a possible regulation of macro- and chaperone-mediated autophagy. Nevertheless, autophagy as a possible mode of action of Montelukast would certainly extend the use of this drug to many degenerative diseases that involve dysregulation of autophagy.

## Electronic Supplementary Material

ESM 1 (TIFF 2702 kb)

high resolution image (PNG 261 kb)

ESM 2 (TIFF 2702 kb)

high resolution imge (PNG 376 kb)

ESM 3 (DOCX 13 kb)

ESM 4 (PDF 1224 kb)

ESM 5 (PDF 1224 kb)
