# Supplementary figures and images for: The Leukotriene Receptor Antagonist Montelukast Reduces Alpha-Synuclein Load and Restores Memory in an Animal Model of Dementia with Lewy Bodies
Source: Neurotherapeutics. 2020 Feb 18;17(3):1061–74. doi: 10.1007/s13311-020-00836-3 (PMC7609773; doi:10.1007/s13311-020-00836-3)

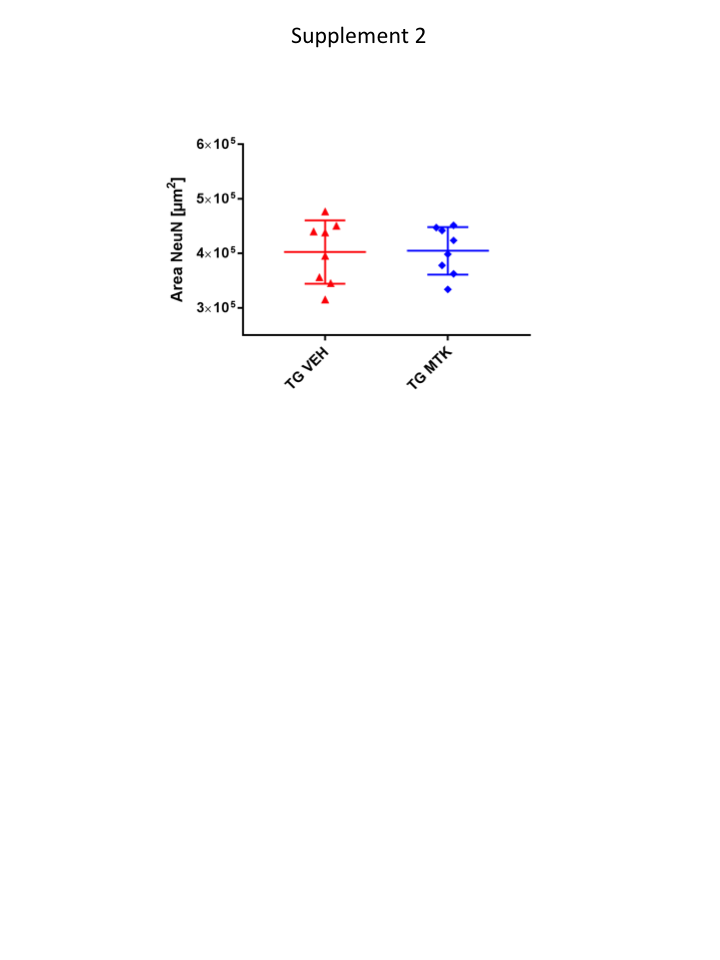

Supplement: Supplementary file 1 — (TIFF 2702 kb) [file 13311_2020_836_MOESM1_ESM.tiff]

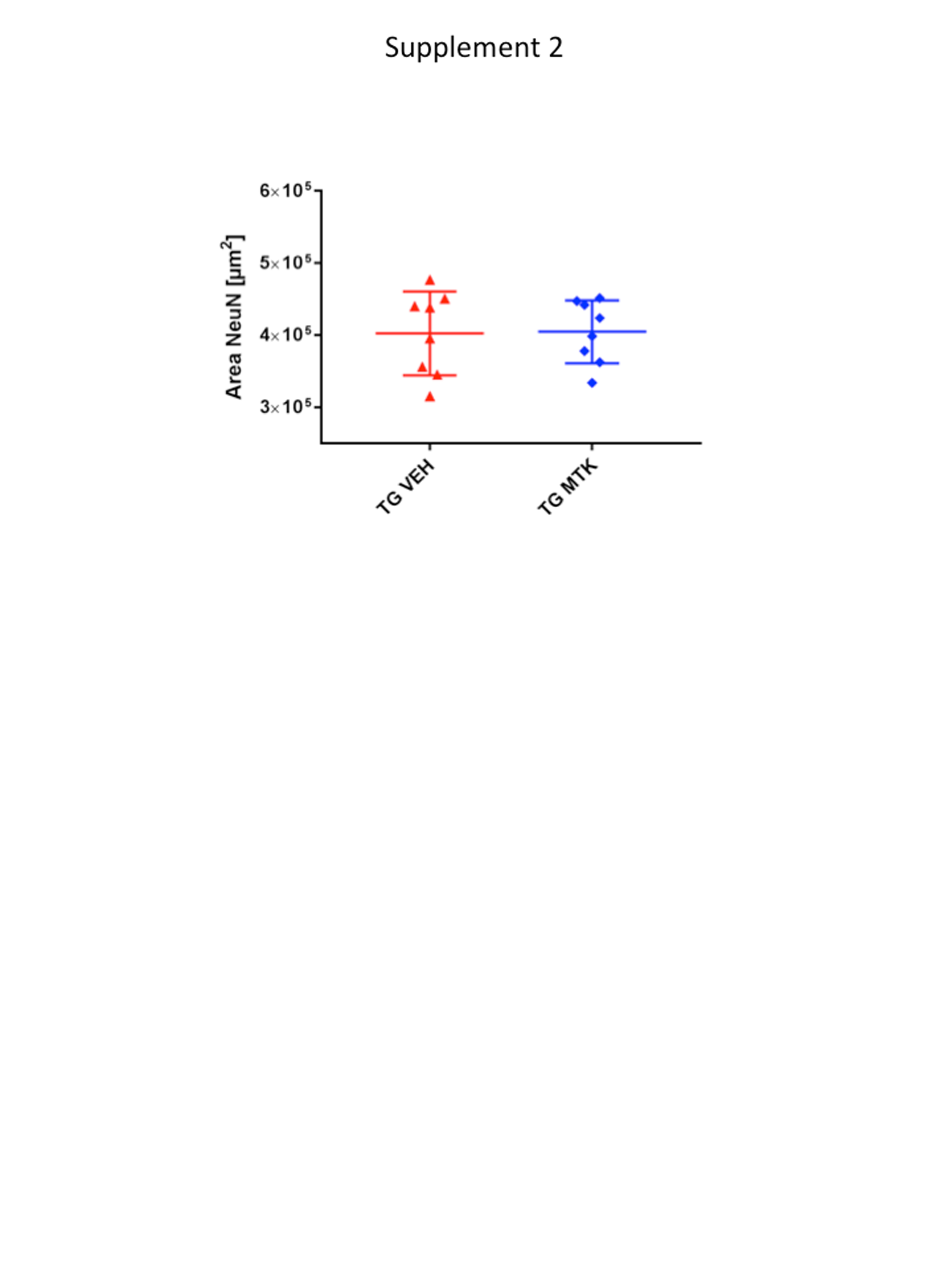

Supplement: Supplementary file 2 — high resolution image (PNG 261 kb) [file 13311_2020_836_Fig6_ESM.png]

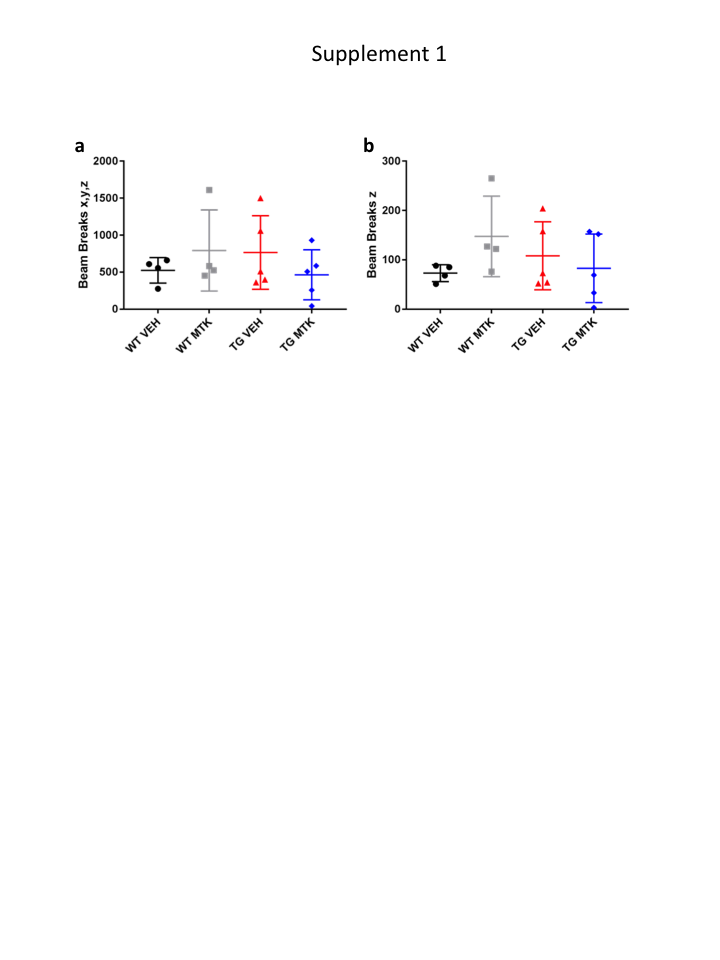

Supplement: Supplementary file 3 — (TIFF 2702 kb) [file 13311_2020_836_MOESM2_ESM.tiff]

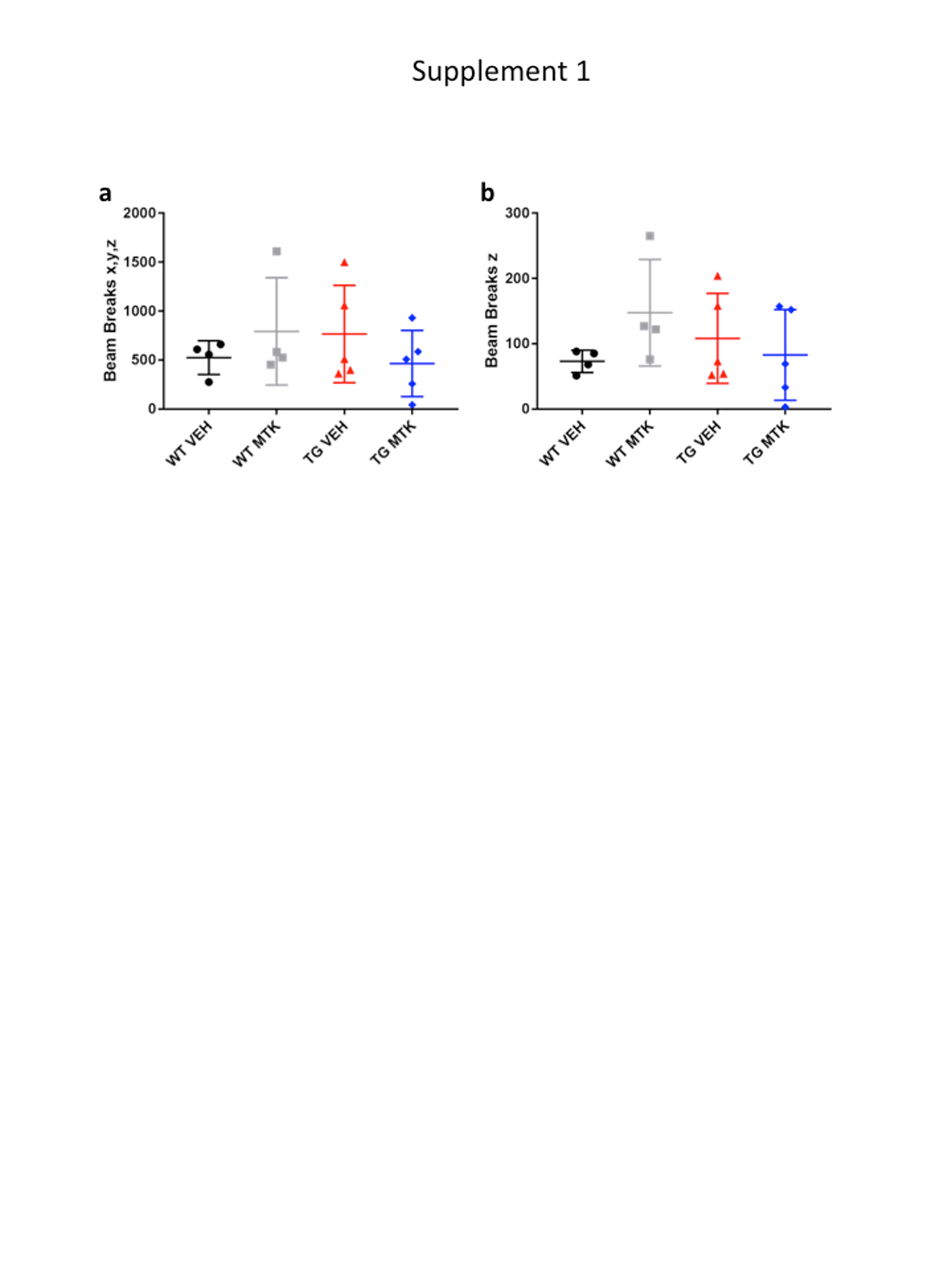

Supplement: Supplementary file 4 — high resolution imge (PNG 376 kb) [file 13311_2020_836_Fig7_ESM.png]
