# Supplementary material for: The Leukotriene Receptor Antagonist Montelukast Reduces Alpha-Synuclein Load and Restores Memory in an Animal Model of Dementia with Lewy Bodies
Source: Neurotherapeutics. 2020 Feb 18;17(3):1061–74. doi: 10.1007/s13311-020-00836-3 (PMC7609773; doi:10.1007/s13311-020-00836-3)
Supplement: Supplementary file 5 — (DOCX 13 kb) [file 13311_2020_836_MOESM3_ESM.docx]

**Supplementary Figure 1:** Spontaneous locomotor function was assessed by analyzing the number of beam breaks in a home cage. The 7.5 months old TG animals did not differ from WT animals in total activity motions (TAM) (a) and rearing (b). Montelukast did not affect these parameters. Data are shown as mean ± SD. *P<0.05, **P<0.01, ***P<0.001. One-way ANOVA followed by Tukey’s post hoc test was performed.

**Supplementary Figure 2:** The area of NeuN+ neurons in the dentate gyrus was measured as a parameter for the dentate gyrus granule cell layer thickness. This parameter did not differ in Montelukast and vehicle treated TG animals. Data are shown as mean ± SD. *P<0.05, **P<0.01, ***P<0.001. One-way ANOVA followed by Tukey’s post hoc test was performed.
